# Effects of sedimentation on soil physical and chemical properties and vegetation characteristics in sand dunes at the Southern Dongting Lake region, China

**DOI:** 10.1038/srep36300

**Published:** 2016-11-03

**Authors:** Ying Pan, Hao Zhang, Xu Li, Yonghong Xie

**Affiliations:** 1 School of Ecology and Environmental Sciences, Yunnan University, Kunming 650031, China; 2 School of Life Science, Yunnan Normal University, Kunming 650500, China; 3 Dongting Lake Station for Wetland Ecosystem Research, Institute of Subtropical Agriculture, The Chinese Academy of Sciences, Changsha 410125, China; 4 Key Laboratory of Agro-ecological Processes in Subtropical Region, Institute of Subtropical Agriculture, The Chinese Academy of Sciences, Hunan 410125, China

## Abstract

Sedimentation is recognized as a major factor determining the ecosystem processes of lake beaches; however, the underlying mechanisms, especially in freshwater sand dunes, have been insufficiently studied. To this end, nine belt transects from nine freshwater sand dunes, classified into low (<23.7 m), medium (25.4–26.0 m), and high-elevation groups (>28.1 m) based on their elevations in 1972, were sampled to investigate differences in sedimentation rate and soil and vegetation characteristics in Southern Dongting Lake, China. Sedimentation rate, soil sand content, and soil pH increased, whereas soil clay, fine silt, moisture (MC), organic matter (OM), total N, and total K content, in addition to the growth and biodiversity of sand dune plants generally decreased with decreasing belt transect elevation. Regression analyses revealed that the negative effects of sedimentation on the ecosystem functions of sand dunes could be attributed to higher fine sand content in deposited sediments and stronger inhibition of plant growth. These results are consistent with previous studies performed in coastal sand dunes, which highlights the importance of sedimentation in determining ecological processes.

Lake beaches are important components of lake ecosystems, which play essential roles in the maintenance of ecosystem functions such as biodiversity conservation, environmental protection, and water storage^1^,^2^. Lake beaches occur in two types of landscapes: lake shores and sand dunes. Lake shores are water-level fluctuation zones between the inland area and the water body^3^. Sand dunes, formed by the deposition of sand carried in waters, are usually located around river mouths of a lake. Extensive studies have been conducted on ecological processes in lake shores^4^,^5^,^6^. However, studies on sand dunes are few.

Sand dune development is a complex ecological process and is related to many factors, especially sedimentation^1^,^7^. Sedimentation, a recurrent event in sand dunes, has a far-reaching effect on soil physical and chemical properties^2^,^7^. For example, the concentrations of fine particles (clay plus silt) and organic matter (OM) usually decrease with increasing sedimentation rate because of extremely low concentrations of fine particles and OM in fresh sediments originating from lakes compared to that in old soils^1^,^8^. Decreased fine particle concentration subsequently leads to a high rate of nutrient leaching in soils^5^,^9^. In addition, sedimentation reduces the amplitude of the daily temperature fluctuations in deep soils and alters soil pH depending on the acidity of the deposited sediments^4^,^10^.

Sedimentation may further affect the growth conditions of sand dune plants because of altered soil physical and chemical properties^11^. Generally, heavy sedimentation is harmful to the growth of almost all plant species due to low oxygen and light availability in buried soils^12^. As a result, only a few sedimentation-tolerant species can successfully colonize under heavy sedimentation. However, slight sedimentation may not inhibit or even stimulate the growth of some sand dune species because the deposited sediments can reduce water evaporation and temperature fluctuations^11^,^13^. Therefore, vegetation characteristics will differ depending on the sedimentation rate in sand dunes.

Additionally, changes in vegetation conditions can exert profound feedback effects on beach soils^14^,^15^. The existence of beach plants is critical for the improvement of soil physical and chemical properties, through weathering of rocks and minerals, release of chemicals and nutrients (e.g., acidic, organic compounds and soil nutrients mainly from the roots and litters), and retention and fixing of fine, organic and mineral particles^14^,^16^. Generally, these positive effects are directly dependent on the growth conditions of beach plants, particularly at the early stage of sand dune development.

In the same drainage basin, low-elevation regions usually retain more sediments than high-elevation regions because of longer waterlogging time^17^,^18^. Consequently, low-elevation sites may contain higher proportions of sand particles in soils, thereby limiting their capacity to conserve moisture (MC), OM and nutrients (e.g., total P, total N, and total K). Moreover, the growth capacity of sand dune plants (including biomass and biodiversity) will be greatly inhibited. With increasing elevation, the sedimentation rate will gradually decrease, and as a result, the growth capacity of sand dune plants and their positive feedback effects on soil structure and soil fertility may gradually increase. Although considerable studies have focused on exploring the developmental processes of lake beaches^19^,^20^, the underlying mechanisms, especially those related to the effects of sedimentation on beach soil and plants, have not been satisfactory explained^21^,^22^.

To test the above-mentioned effects of sedimentation on soil and vegetation characteristics in sand dunes, nine belt transects from nine different sand dunes (Fig. 1) were chosen from Southern Dongting Lake, China. Sand dunes are very active in Dongting Lake, where a large amount of sediment (about 1.48 × 10^8^ t yr^−1^) is deposited annually during flooding time^23^, providing an ideal site for the study of the relationships among sedimentation rate, soil properties, and vegetation characteristics. We predicted that: 1) the sedimentation rate might be the highest in low-elevation belt transects that would inhibit the improvement of both soil and vegetation characteristics, resulting in the lowest content of fine particles (clay and silt), MC, OM, and nutrients (including total N, total P, and total K) in sand dune soils, and the lowest values in plant biomass and biodiversity; 2) the sedimentation rate may gradually decrease with increasing elevation, and therefore, the growth capacity of sand dune plants and their positive feedback effects on soil structure and soil fertility would gradually increase, which in turn would lead to increases in plant biomass and biodiversity, as well as the contents of fine particles, MC, OM, and nutrients in sand dune soils.

## Results

### Sedimentation rate

Sedimentation rate differed significantly among the three belt transect groups, which was the highest in the low-elevation belt transects, intermediate in the medium-elevation belt transects, and lowest in the high-elevation belt transects (*p* = 0.001, Table 1).

### Vegetation characteristics

Sixty-seven vascular plants belonging to 57 genera and 26 families were identified in the 75 quadrats. The family *Gramineae* had the highest species number, followed by *Compositae* and *Lamiaceae*. Vegetation characteristics varied considerably with changes in elevation. Plant biomass was significantly lower (*p* = 0.034) in belt transects with low elevation (1.79 ± 0.5 kg m^−2^) than in those with medium (6.51 ± 1.42 kg m^−2^) and high elevations (6.32 ± 1.08 kg m^−2^), and the Shannon-Wiener index generally increased with increasing elevation (from 1.4 to 2.5, *p* = 0.032, Fig. 2). *Phalaris arundinacea* (with an IV of 41.2% ± 21.1%) and *Oenanthe benghalensis* (IV of 27.9% ± 27.4%) were the predominant species in the low-elevation belt transects. *P. arundinacea* was replaced by *Miscanthus sacchariflorus* (IV of 45.4% ± 4.7%) in the medium-elevation belt transects, and by *M. sacchariflorus* (IV of 38.3% ± 9.6%) and *Phragmites communis* (IV of 14.2% ± 1.9%) in the high-elevation belt transects (Table 2).

### Physical characteristics of sand dune soils

Soil MC was lower (20.04%) in belt transects at low elevation than in those at medium and high elevations (24.43–26.73%, *p* = 0.036, Fig. 3), especially at the 0–20 cm soil layer (*p* = 0.042).

With increasing elevation, the content of fine sand decreased from 63.9% to 14.7%, whereas the content of fine silt and clay increased from 20.2% to 49.0%, and from 6.4% to 24.1%, respectively, in sand dune soils (*p* < 0.05, Table 3). Moreover, the increasing trends for fine silt and clay with increasing elevation were significant in all three soil layers (*p* < 0.05).

### Chemical characteristics of sand dune soils

There were significant differences in soil pH, OM, total N and total K among the three belt transects groups at the three different elevations (*p* < 0.05, Table 3). With increasing elevation, pH decreased from 8.76 to 8.58 (*p* = 0.01), whereas the content of OM, total K, and total N increased from 0.67% to 1.62%, from 13.02 g kg^−1^ to 20.99 g kg^−1^, and from 0.082% to 0.109%, respectively (*p* < 0.05). However, total P did not differ significantly among the three belt transect groups (*p* > 0.05).

The vertical distribution of soil OM differed significantly across the three elevation groups. In the low-elevation belt transects, soil OM was lower in the 0–20 m soil layer than in the 40–60 cm soil layer (0.42% versus 0.95%, *p* = 0.017). In contrast, in the high-elevation belt transects, soil OM was higher in the 0–20 cm layer than in the 20–40 cm and 40–60 cm soil layers (2.01% versus 1.38–1.48%, *p* = 0.006). OM continuously accumulated in sand dune soils with increasing elevation, especially in the surface-layer soils.

### Relationships among sedimentation rate, soil properties, and vegetation characteristics

Linear regression analysis revealed that sedimentation rate was significantly and positively associated with fine sand content (*p* = 0.008, Fig. 4A). In addition, fine sand content was significantly and negatively associated with MC (*p* = 0.001, Fig. 4B), OM (*p* = 0.000, Fig. 4C), total N (*p* = 0.002, Fig. 4D) and total K (*p* = 0.012, Fig. 4E), but positively with pH (*p* = 0.025, Fig. 4F). Moreover, pH was negatively associated with OM (*p* = 0.019, Fig. 4G).

Plant biomass (*p* = 0.000, Fig. 5A) and the Shannon-Wiener index (*p* = 0.027, Fig. 5B) were significantly and negatively associated with sedimentation rate. Moreover, plant biomass showed significantly positive relationships with the fine silt content (*p* = 0.013, Fig. 5C), MC (*p* = 0.008, Fig. 5D), total K (*p* = 0.024, Fig. 5E), and OM (*p* = 0.011, Fig. 5F, R^2^ ranging between 0.51 and 0.66).

## Discussion

The main objective of this study was to investigate the effect of sedimentation on the ecological processes in freshwater sand dunes, which is essential for understanding the development process of the sand dune ecosystems^24^. Our study has shown that sedimentation influences the ecological processes of sand dunes by direct effects on soil and vegetation characteristics and indirect effects through modulation of the ameliorating effects of vegetation on soil properties.

Sedimentation is often disadvantageous to the improvement of soil structure and soil fertility in freshwater sand dunes. First, sedimentation limits the improvement of soil structure because the deposited fresh sediments usually have a high proportion of coarse particles (fine and/or coarse sand) compared to that in old soils, such as in Dongting Lake, where coarse particles account for more than 50% of all particles in deposited sediments^1^. The high proportion of coarse particles will subsequently lead to a reduction in the ability of sand dune soil to conserve water, OM, and nutrients^5^,^9^,^25^. Consistently, in the present study, sedimentation rate was significantly and positively associated with the soil fine sand content; moreover, the soil fine sand content was significantly and negatively correlated with soil OM, total N, and total K, respectively (Fig. 4). Therefore, the lowest contents of soil clay, fine silt, MC, OM, total N, and total K in the low-elevation belt transects might be attributed to the highest sedimentation rates in these areas. In addition, soil pH was significantly higher in the belt transects at low and medium elevations than in those at high elevation. This effect might be attributed to: 1) deposited sediments, which are usually alkaline in Dongting Lake^26^, and 2) extremely low OM content. Limited decomposition of OM will prevent the synthesis of carboxylic groups, which may decrease soil pH after dissociation^27^, as indicated by the significant negative correlation between OM and pH in the present study (Fig. 4G).

Development of sand dune vegetation could also be influenced by sedimentation. A high rate of sedimentation inhibits the growth and colonization of all but highly specialized species^13^, which might be attributed to the low availability of oxygen and light in buried soils^4^,^11^,^13^. In this study, only a few sedimentation-tolerant species were able to successfully colonize (e.g. *Phalaris arundinacea* and *Alternanthera philoxeroides*, Table 2), and plant biomass and biodiversity (Shannon-Wiener index, Fig. 2) were lowest in the low-elevation belt transects with the highest sedimentation rate. The negative effects of sedimentation on vegetation characteristics were demonstrated by the significant negative correlations between sedimentation with plant biomass and Shannon-Wiener index (Fig. 5A,B). All of these results generally support our first prediction that the sedimentation rate might be highest in the low-elevation belt transects, and thus would lead to the lowest contents of fine particles, MC, OM, and nutrients in sand dune soil, as well as the lowest values of plant biomass and biodiversity. Subsequently, plant growth and biodiversity greatly improved in the medium- and high-elevation belt transects as a result of decreasing sedimentation rate.

Enhancement of the growth conditions of sand dune plants may subsequently facilitate improvement in soil structure and soil fertility. Plants can release a variety of organic compounds from their roots and increase litter fall mass, which may benefit the formation of soil aggregated structure and enhance soil fertility (OM and nutrients)^15^,^28^. Therefore, soil physical and chemical properties will gradually be improved with increasing plant biomass during the primary succession of beach communities. These predictions are supported by our study, as there were significant positive correlations between plant biomass with the contents of fine silt, MC, total K, and OM (Fig. 5). Additionally, plant biodiversity increased significantly with increasing elevation, which may also be important in the amelioration of soil structure and soil fertility. The mechanism might be related to an increase in primary production with increasing plant biodiversity in the early stages of sand dune development because different plants typically occupy different niches and/or facilitate each other’s acclimation to environmental stresses^29^. Plant roots and litter fall mainly distribute in the surface of soils^30^, and the accumulation of soil OM and nutrients may be more obvious in the surface soil layer^31^,^32^, which is consistent with the findings of the present study. These results support our second prediction that the growth capacity (growth and biodiversity) of sand dune plants will gradually improve, and the beneficial effects of plants on soil structure and fertility will gradually increase with increasing elevation. Surprisingly, total P was unaffected by the increase in elevation, probably because the contents of total P are extremely low in Dongting Lake^33^.

In summary, this study clearly describes the effects of sedimentation on the ecological process in sand dunes. Briefly, the conditions of soil MC, OM, nutrients, and pH were poor and the growth of sand dune plants was severely inhibited in the low-elevation belt transects because they had the highest sedimentation rate. Subsequently, the sedimentation rate gradually decreased with increasing belt transect elevation. As a result, plant growth and biodiversity improved, which gradually improved soil structure and soil fertility conditions. To date, the influence of sedimentation on sand dune dynamics has received much attention, which focus on aeolian sand deposition in coastal sand dunes^34^. However, less attention has been paid to freshwater sand dunes where the deposited sediments were carried by water flow. Our results demonstrated that sedimentation plays an important role in determining the dynamics of freshwater sand dunes through modulating the beneficial effects of vegetation on soil properties. Our research focused on a simple approach to explore the dynamics of soil and vegetation; however, other important environment factors such as hydrology conditions, shade, and drought were not included in this study. Thus, studies are required to gain a comprehensive understanding of all factors affecting sand dunes in the lake ecosystem^4^,^35^.

## Materials and Methods

### Study site

Dongting Lake (28°43′–29°32′N, 112°54′–113°8′E), the second largest freshwater lake in China and a typical river-connected lake, is located in the south bank of the middle reach of the Yangtze river. Dongting Lake is primarily composed of three sub-lakes: the Eastern, Southern, and Western Dongting Lakes^36^. Sedimentation is a recurrent event in the sand dunes of Dongting Lake. Large amounts of sediments are carried by water flow and then deposited on sand dune surfaces as a result of reduced flow velocity in these areas, especially during flooding seasons^17^,^37^. Of these lakes, the Southern Dongting Lake (28°54′–28°55′N, 112°28′–112°35′E) has the highest mean sedimentation rate of approximately 19.11 mm per year^38^. It receives silt from “three channels” (Songzi, Taiping and Ouchi) connected with the Yangtze river, and from “four rivers” (the Xiang, Zi, Yuan and Li rivers) of Hunan Province. In Southern Dongting Lake, active sedimentation favors the formation and expansion of sand dunes, which exerts a far-reaching influence on plant maintenance and evolution^1^.

### Site selection and sampling

**Figure 1:**
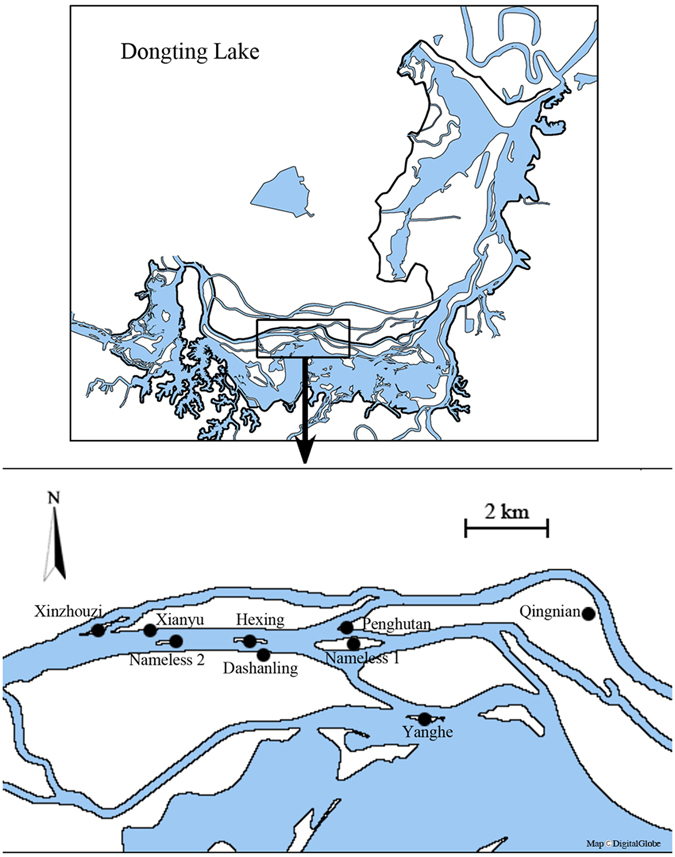
Schematic map of sand dunes sampled along Southern Dongting Lake, China. Figure 1 was created using Adobe Photoshop 7.0 (Map data: Google, DigitalGlobe).

**Figure 2:**
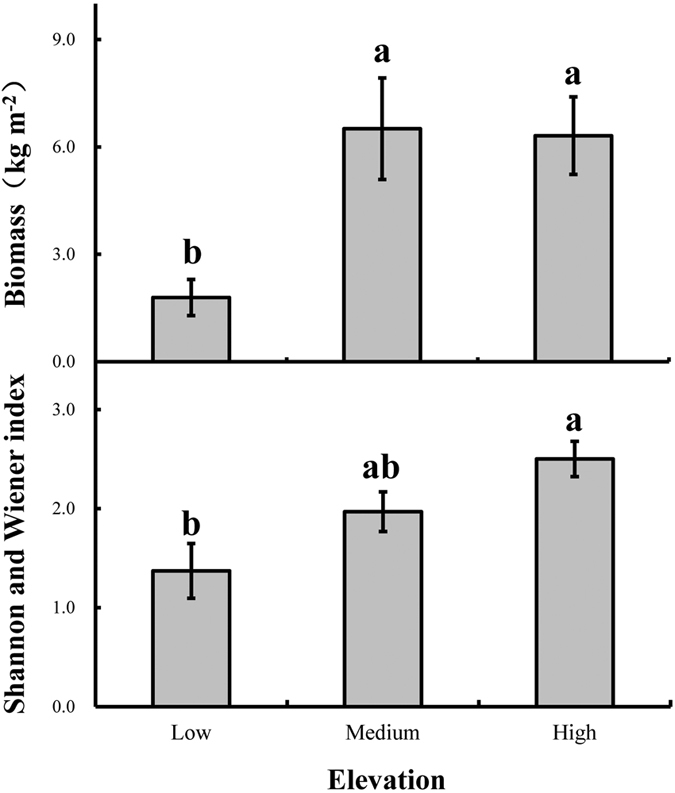
Variations (means ± 1 s.e., n = 3) in biomass (kg m^−2^) and Shannon-Wiener index in belt transects under three different elevation conditions. Different letters indicate significant differences among the three different elevation conditions (*p* < 0.05). Figure 2 was created using Microsoft Excel 2010.

**Figure 3:**
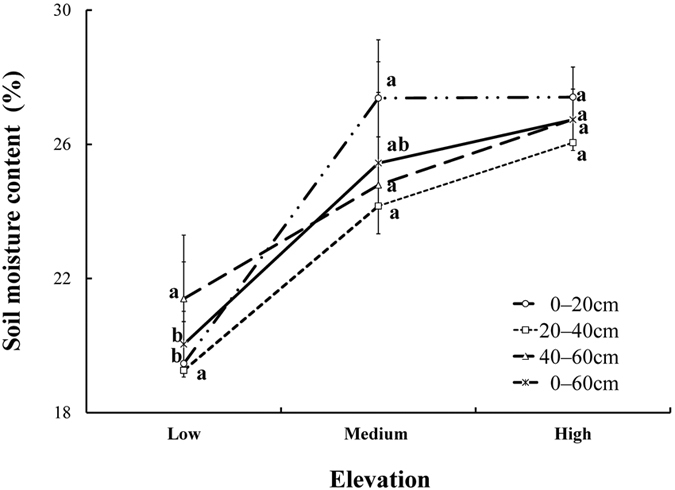
Variation in soil moisture content (%) under three different elevation conditions in the 0–20 cm, 20–40 cm, 40–60 cm and 0–60 cm soil layers of sand dunes along Southern Dongting Lake, China. Results are expressed as the mean ± 1 s.e. (n = 3). Different letters indicate significant differences among the three different elevation conditions (*p* < 0.05). Figure 3 was created using Microsoft Excel 2010.

**Figure 4:**
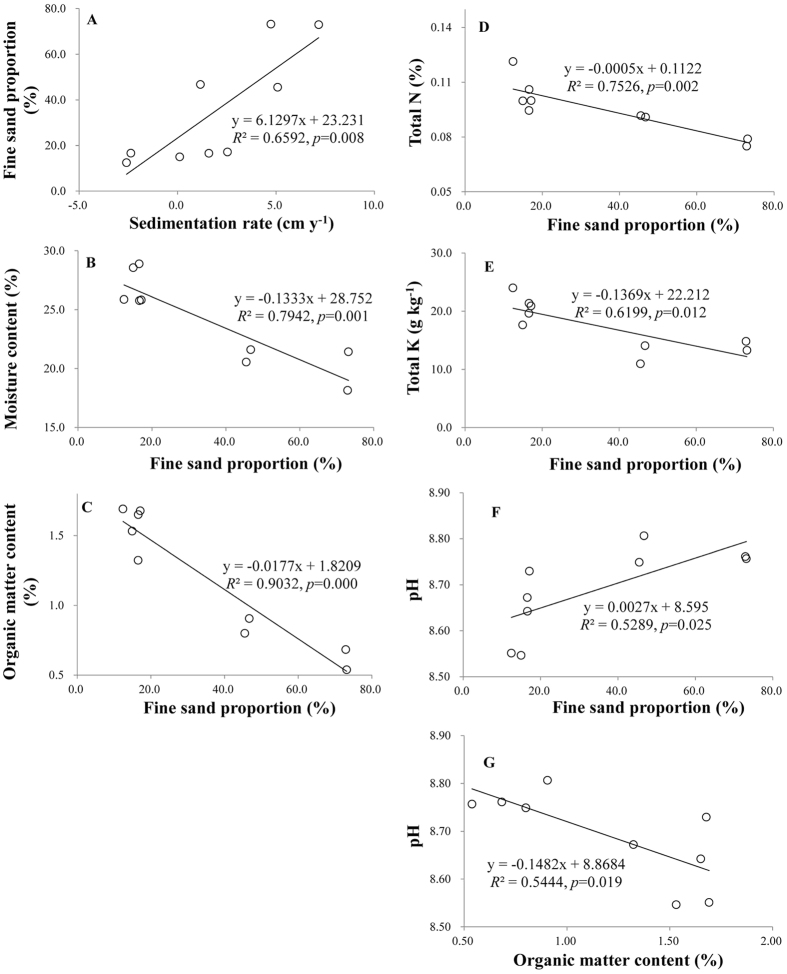
Relationships between sedimentation rate and soil physical and chemical properties; df = (1,8). Relationships between (**A**) sedimentation rate and fine sand proportion; (**B**) fine sand proportion and moisture condition; (**C**) fine sand proportion and organic matter content; (**D**) fine sand proportion and total N; (**E**) fine sand proportion and total K; (**F**) fine sand proportion and pH; and (**G**) organic matter content and pH. Figure 4 was created using Microsoft Excel 2010.

**Figure 5:**
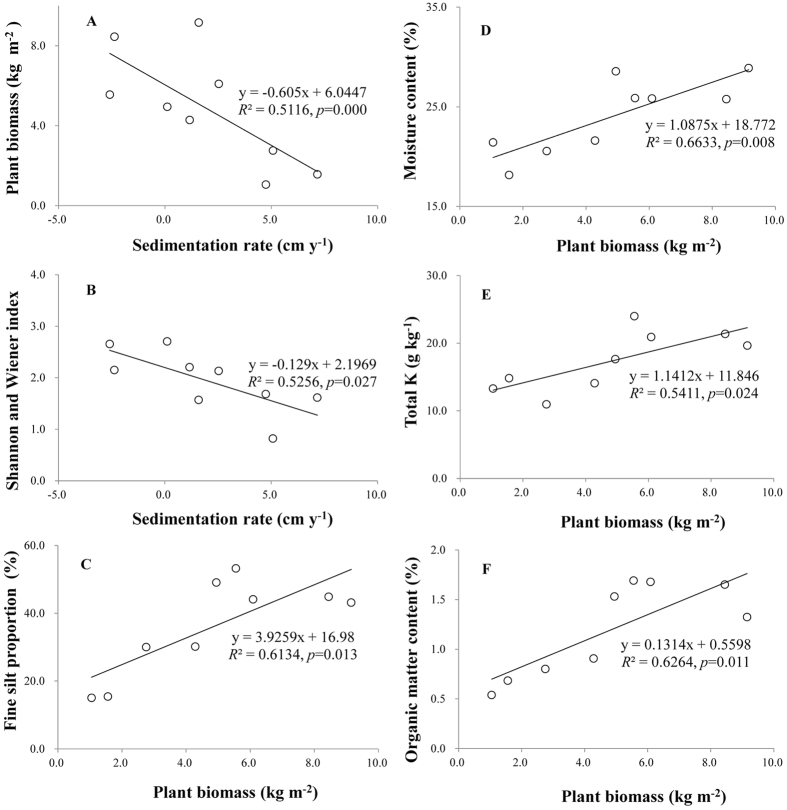
Relationships among sedimentation rate, vegetation characteristics and soil properties, df = (1,8). Relationships between (**A**) sedimentation rate and plant biomass; (**B**) sedimentation rate and Shannon and Wiener index; (**C**) plant biomass and fine sand proportion; (**D**) plant biomass and moisture content; (**E**) plant biomass and total K; (**E**) plant biomass and organic material content. Figure 5 was created using Microsoft Excel 2010.

Field investigations were conducted at nine belt transects (the belt transect was in the middle elevation site of each sand dune) from nine sand dunes in Southern Dongting Lake in April 2009, which is the later period of the non-flooding season (Fig. 1). These nine belt transects were classified into three groups (Table 1) based on their early elevation in 1972 indicated by an elevation map (which used the Yellow Sea as the datum plane) released by the Yangtze River Water Conservancy Commission (Table 1). The groups were as follows: low (<24.4 m), medium (25.3–26.0 m), and high-elevation belt transects (>28.1 m). As the belt transect was distributed in the middle elevation site of each sand dune, the above three sand dune groups could also represent three developmental stages, i.e., the early-, medium-, and the later-developmental stage of sand dunes, respectively. The formation times of these three sand dune groups spanned at least 20 years according to our investigation, which we consider to have been sufficiently long for us to investigate the relationships among sedimentation, and soil and plant characteristics in these active environments^39^. Two or three sample plots (10 m × 10 m) were selected in each belt transect according to dune area. Twenty-five plots were investigated in total. Soil samples were collected using a 5 cm diameter core using the multi-point sampling method^40^, and the soil cores were divided into three layers: 0–20 cm, 20–40 cm, and 40–60 cm. At each plot, three quadrats (1 m × 1 m) were randomly set for evaluation of vegetation. All soil samples were collected away from *Populus euramericana* trees (an alien tree species introduced into the area by the local people as raw material for paper-making), because *P. euramericana* might alter the inherent characteristics of sand dunes.

### Sedimentation rate

The sedimentation rate (SR) of each sample plot was calculated by the following formula:


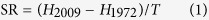


where *H*_*2009*_ (taken with a Barigo altimeter) and *H*_1972_ (determined based on an elevation map released by the Yangtze River Water Conservancy Commission) are the elevation of the sample plot in 2009 and 1972, respectively (see Table 1), and *T* is the sedimentation time from 1972 to 2009 (i.e., 37 years).

### Vegetation characteristics

Plant species, above-ground fresh biomass (including living tissues and standing dead tissues), and plant height were recorded for each quadrat. Individuals (including ramets) of each species were separated and counted, and the plant density of each species was calculated as the number of individuals per square meter. Canopy coverage of each species was calculated as the ratio of the total canopy area to quadrat size. Finally, the importance value index (IV) and plant biodiversity were calculated based on the above parameters as follows:


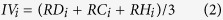



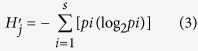


where *IV*_*i*_ indicates the IV of the *i*th species, and *RD*_*i*_, *RC*_*i*_ and *RH*_*i*_ are the relative density, relative coverage, and relative height of the *i*th species, respectively^41^. 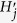 is the Shannon-Wiener index for species diversity, and *p*_*i*_ is the ratio of the IV of the *i*th species to the IV of the entire community of the *j*th plot^42^.

### Soil properties

Soil properties were analyzed separately for the 0–20, 20–40 and 40–60 cm soil layers, which were collected as described earlier. For analysis of the soil properties, soil samples were first air-dried and sieved to remove coarse fragments (the sieves used were <2 mm for all analyses; in particular, analyses for total N and for total K and P used <0.25 mm and <0.149 mm sieves, respectively). MC was measured as the difference in the weight of fresh subsamples before and after incubation at 80 °C for 24 h. Soil particle composition was classified into clay (<0.002 mm), fine silt (0.002–0.02 mm), coarse silt (0.02–0.05 mm), fine sand (0.05–0.25 mm) and coarse sand (0.25–2 mm) based on particle diameter^43^, using a laser particle size analyzer (Mastersizer 2000). Soil pH was measured using a digital pH meter to estimate the pH of 10.0-g of soil suspended in 25.0 ml of a 0.01 mol L^−1^ CaCl_2_ solution. OM content was determined using the wet digestion method for potassium dichromate oxidation^44^. Total soil N was determined using the Kjeldahl method^45^. Total P was analyzed by dissolving soil samples in NaOH solution followed by molybdenum-stibium colorimetry. Total K was measured using the NaOH melting-flaming luminosity method^46^.

### Statistical analysis

Data from 75 soil samples and 67 vascular species were analyzed. Prior to analysis, all data were tested for homogeneity of t variance using the Levenes test. Soil and vegetation characteristics from two or three plots in each belt transect were averaged. The significance of differences in soil physical and chemical properties under the three elevation conditions was estimated using Tukey’s test at a 0.05 significance level. Linear regression analysis was used to determine the relationships between the continuous variables. All statistical analyses were performed using the SPSS 13.0 software package (SPSS Inc., Chicago, USA).

## Additional Information

**How to cite this article**: Pan, Y. *et al*. Effects of sedimentation on soil physical and chemical properties and vegetation characteristics in sand dunes at the Southern Dongting Lake region, China. *Sci. Rep.* **6**, 36300; doi: 10.1038/srep36300 (2016).

**Publisher’s note:** Springer Nature remains neutral with regard to jurisdictional claims in published maps and institutional affiliations.

## Figures and Tables

**Table 1:** Characteristics of sampling sites in belt transects along the South Dongting Lake, China.

Elevation	Belt transect	Plot number	Location	Elevation in 1972 (m)	Elevation in 2009 (m)	Mean sedimentation rate (cm y^−1^)
Low	Xinzhouzi	1, 2	N28°55′18.9″ E112°28′07.4″	23.57 ± 0.51	25.33 ± 0.05	5.67 ± 0.66a
	Nameless 2	3, 4, 5	N28°55′12.7″ E112°29′51.9″	23.68 ± 0.21	26.33 ± 0.45	
	Hexing	6, 7	N28°55′09.8″ E112°30′30.0″	24.41 ± 0.37	26.30 ± 0.79	
Medium	Yanghe	8, 9, 10	N28°54′05.7″ E112°33′24.0″	25.97 ± 0.06	26.56 ± 0.52	1.76 ± 0.35b
	Nameless 1	11, 12, 13	N28°55′12.8″ E112°28′13.8″	25.65 ± 0.23	26.08 ± 0.24	
	Penghutan	14, 15, 16	N28°55′18.5″ E112°32′03.5″	25.34 ± 0.08	26.28 ± 0.19	
High	Qingnian	17, 18, 19	N28°55′28.3″ E112°35′54.7″	28.98 ± 0.03	29.02 ± 0.03	−1.62 ± 0.87c
	Dashanling	20, 21, 22	N28°55′01.5″ E112°30′50.6″	29.21 ± 0.23	28.34 ± 0.55	
	Xianyu	23, 24, 25	N28°55′21.1″ E112°29′18.7″	28.13 ± 0.78	27.17 ± 1.01	

Results are expressed as the mean ± 1SE. Different letters (a–c) indicate significant differences in sedimentation rate among the three different elevation conditions (*p* < 0.05). Elevation data for 2009 were determined using a Barigo altimeter, and data for 1972 were based on an elevation map released by the Yangtze River Water Conservancy Commission.

**Table 2:** Species composition and the importance value index (IV) of the dominant species in sand dunes under the three elevation conditions studied.

Elevation	Dominant Species	IV (%)	n
Low	*Phalaris arundinacea*	41.2 ± 21.1	3
	*Oenanthe benghalensis*	27.9 ± 27.4	3
	*Miscanthus sacchariflorus*	14.2 ± 0.9	3
	*Hemarthria compressa*	8.5 ± 8.5	3
	*Phragmites communis*	4.1 ± 0.4	3
	*Alternanthera philoxeroides*	1.7 ± 1.7	3
Medium	*Miscanthus sacchariflorus*	45.4 ± 4.7	3
	*Oenanthe benghalensis*	31.1 ± 2.4	3
	*Portulaca grandiflora*	5.7 ± 5.7	3
	*Hemarthda compressa*	3.6 ± 3.6	3
	*Trigonotis peduncularis*	2.6 ± 2.6	3
	*Phragmites communis*	1.5 ± 1.5	3
	*Herba Paederiae*	1.4 ± 0.8	3
High	*Miscanthus sacchariflorus*	38.3 ± 9.6	3
	*Oenanthe benghalensis*	14.5 ± 10.3	3
	*Phragmites communis*	14.2 ± 1.9	3
	*Polygonum hydropiper*	11.2 ± 5.2	3
	*Portulaca grandiflora*	4.8 ± 3.5	3
	*Humulus scandens*	4.3 ± 4.3	3
	*Cardamine* spp.	2.6 ± 1.3	3
	*Mentha haplocalyx*	1.7 ± 1.7	3
	*Hemarthda compressa*	1.2 ± 1.2	3
	*Hemistepta lyrata*	1.2 ± 0.8	3
	*Carex* spp.	1.2 ± 1.2	3

Companion species are listed according to their IV from large to small and values under 1% are not reported. Results are expressed as the mean ± 1s.e. (n = 3).

**Table 3:** Mean values of soil particle composition, pH, organic matter (OM), total N, total P, and total K of soil from sand dunes under the three elevation conditions studied.

	Soil properties	Low elevation	Medium elevation	High elevation
Soil particle composition	Clay <0.002 mm (%)	6.4 ± 1.8b	18.4 ± 2.9a	24.1 ± 2.9a
	Fine silt 0.002–0.02 mm (%)	20.2 ± 4.9b	39.1 ± 4.5a	49.0 ± 2.4a
	Coarse silt 0.02–0.05 mm (%)	8.8 ± 2.6a	15.3 ± 3.0a	12.0 ± 3.6a
	Fine sand 0.05–0.25 mm (%)	63.9 ± 9.2a	26.8 ± 10.0b	14.7 ± 1.2b
	Coarse sand 0.25–2 mm (%)	1.0 ± 0.3a	0.3 ± 0.1a	0.2 ± 0a
Soil chemical properties	pH	8.76 ± 0a	8.74 ± 0.04a	8.58 ± 0.03b
	OM (%)	0.67±0.08b	1.30 ± 0.22a	1.62 ± 0.05a
	Total N (%)	0.082 ± 0.005b	0.095 ± 0.003ab	0.109 ± 0.006a
	Total P (g kg^−1^)	0.061 ± 0.007a	0.063 ± 0a	0.073 ± 0.007a
	Total K (g kg^−1^)	13.02 ± 1.13b	18.21 ± 2.10ab	20.99 ± 1.84a
	n	3	3	3

Results are expressed as the mean ± 1 s.e. (n = 3). Letters (a–c) indicate significant difference among different elevation conditions (*p* < 0.05).
